# Vitamin D receptor gene FokI but not TaqI, ApaI, BsmI polymorphism is associated with Hashimoto’s thyroiditis: a meta-analysis

**DOI:** 10.1038/srep41540

**Published:** 2017-01-30

**Authors:** Xiaofei Wang, Wenli Cheng, Yu Ma, Jingqiang Zhu

**Affiliations:** 1 Department of Thyroid and Breast Surgery, West China Hospital, Sichuan University, Chendu, China; 2 Department of General Surgery, Affiliated Hospital of North Sichuan Medical College, Nanchong, China; 3 Department of Otolaryngology-Head and Neck Surgery, Affiliated Hospital of North Sichuan Medical College, Nanchong, China

## Abstract

Four VD receptor (VDR) gene polymorphisms (*TaqI, ApaI, FokI* and *BsmI*) have been reported to influence Hashimoto’s thyroiditis (HT) risk. However, individual studies have produced inconsistent results. We conducted a comprehensive meta-analysis of eleven case-control studies to better understand roles of the four polymorphisms in HT development. The results showed only *FokI* polymorphism was significantly associated with the risk of HT (F vs f: OR = 1.44, 95% CI = 1.09–1.91, *P* = 0.010; FF vs Ff + ff: OR = 1.72, 95% CI = 1.09–2.70, *P* = 0.019). Subgroup analyses demonstrated the significant effect was only present in Asian population (F vs f: OR = 1.45, 95% CI = 1.07–1.95, *P* = 0.016; FF vs ff: OR = 1.64, 95% CI = 1.03–2.59, *P* = 0.036; FF + Ff vs ff: OR = 1.34, 95% CI = 1.00–1.80, *P* = 0.047; FF vs Ff + ff: OR = 1.64, 95% CI = 1.03–2.64, *P* = 0.039), but not in Caucasian. For *TaqI, ApaI* and *BsmI* polymorphisms, no significant association was found in any model comparison. Based on the current literature, it appears that only VDR *FokI* polymorphism is associated with HT risk in Asian population, but not in Caucasians; and the *TaqI, ApaI* and *BsmI* polymorphisms have not positive association neither in the overall population, nor when stratified by ethnicity. Further well-designed studies with larger sample sizes and different ethnic population are needed to clarify the present findings.

Hashimoto’s thyroiditis (HT) is an autoimmune thyroid disease (AITD), which has been reported to lead hypothyroidism in up to 5% of population^1^,^2^,^3^. It is characterized by diffuse infiltration of chronic lymphocytic cells and presence of high serum thyroid antibodies concentrations^4^,^5^,^6^. Accumulating evidence has demonstrated that HT may be an autoimmune disease triggered by both genetic and environmental factors^7^,^8^,^9^. Data on twins studies showed the concordance rates for HT were significantly higher among monozygotic twins than dizygotic twins^10^,^11^, which suggests that patients with HT have a substantial inherited susceptibility. Moreover, a number of studies have reported certain immunomodulatory genes polymorphisms, such as fork head box P3 (FOXP3), cytotoxic T-lymphocyte-associated protein-4 (CTLA-4) and human leukocyte antigen (HLA) family, were involved in the susceptibility to HT^12^,^13^,^14^,^15^. Thus, HT seems to be a polygenic disease with a complex mode of inheritance. However, the precise gene factors inciting the condition remain not fully comprehended.

Vitamin D receptor (VDR) is a ligand inducible transcription factor, which is harbored on many human immune cells^16^,^17^,^18^. The active vitamin D, an important immunomodulator, exerts its biological effects through binding to the VDR, and in this way to modulate immune cells activity, triggering innate and adaptive immune responses^19^,^20^,^21^. Certain single nucleotide polymorphisms (SNPs) of the VDR gene may modify vitamin D function. More than sixty SNPs of human VDR gene have been reported^22^,^23^. Among them, four common VDR SNPs: TaqI (rs731236, exon 9, +65058 T > C), ApaI (rs7975232, intron 8, +64978 C > A), FokI (rs2228570, exon 2, +30920 C > T) and BsmI (rs1544410, intron 8, + 63980 G > A), were studied intensively for association with various human traits. They were reported to affect the risk of several autoimmune disorders, including rheumatoid arthritis, systemic lupus erythematosus, inflammatory bowel disease, diabetes mellitus and the other AITD (Graves’ diseases, GD)^21^,^24^,^25^,^26^,^27^. Recently, several studies have also investigated the association of the four VDR SNPs and HT susceptibility^28^,^29^,^30^,^31^,^32^,^33^,^34^,^35^,^36^,^37^,^38^, but their results were inconsistent.

Therefore, it is necessary to carry out a meta-analysis of the available evidence to clarify this inconsistency and provide a much comprehensive and quantitative understanding of the association of VDR gene polymorphisms with HT risk.

## Results

### Study characteristics

**Figure 1:**
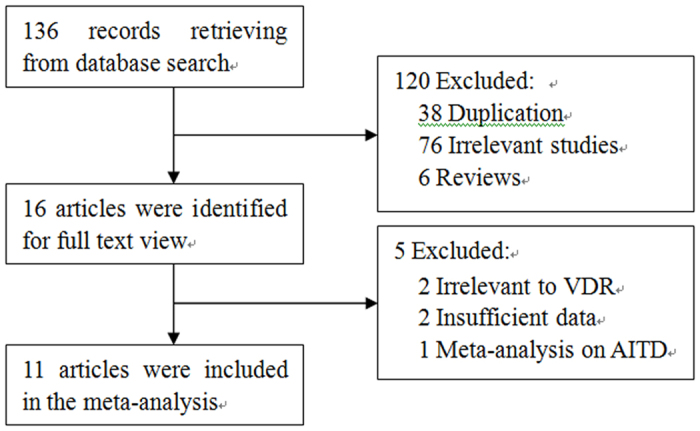
Flow diagram of study selection in this meta-analysis.

As shown in Fig. 1, the search strategy retrieved 136 articles. After further evaluation, only eleven relevant studies^28^,^29^,^30^,^31^,^32^,^33^,^34^,^35^,^36^,^37^,^38^ finally fulfilled the inclusion criteria, including 1338 cases and 1303 controls. All were case-control studies. Nine studies published in English and two in Chinese. There were six studies involving Asians^28^,^29^,^31^,^32^,^34^,^36^, and the other five studies involving Caucasians^30^,^33^,^35^,^37^,^38^. The VDR gene was genotyped by polymerase chain reaction–restriction fragment length polymorphism (PCR-RFLP) in all studies, excepting one study used Matrix assisted laser desorption ionization-time of flight mass spectrometer (MALDI-TOF-MS)^36^. The NOS scores of included studies ranged from 6 to 9 stars, with a median 7 stars. All studies but two^28^,^36^ was scored as high quality studies (≥7 stars). Table 1 summaries the characteristics of these studies. The following 4 VDR SNPs were studied: TaqI (rs731236, alleles T/t), ApaI (rs7975232, alleles A/a), FokI (rs2228570, alleles F/f), and BsmI (rs1544410, alleles B/b). Genotypes are designated conventionally by the first letter of the name of restriction enzymes, with a lower case indicating the presence of restriction site, whereas an upper-case letter indicating its absence. Table 2 shows the genotype distribution in the cases and controls, along with the *P*-value of chi square test for genotype distribution and HWE in control group. HT is often diagnosed mainly on the basis of laboratory and ultrasonographic features, such as positive serum anti-thyroid antibodies, heterogeneous echo-structure with diffuse or patchy hypoechogenicity at ultrasonography, with hypothyroid or euthyroid metabolic state.

### Meta-analysis results

Table 3 provides the pooled results regarding the association of the four VDR gene polymorphisms and HT risk under five different genetic models, along with the *P*-value of Egger’s test for publication bias.

### *FokI* polymorphism

**Figure 2:**
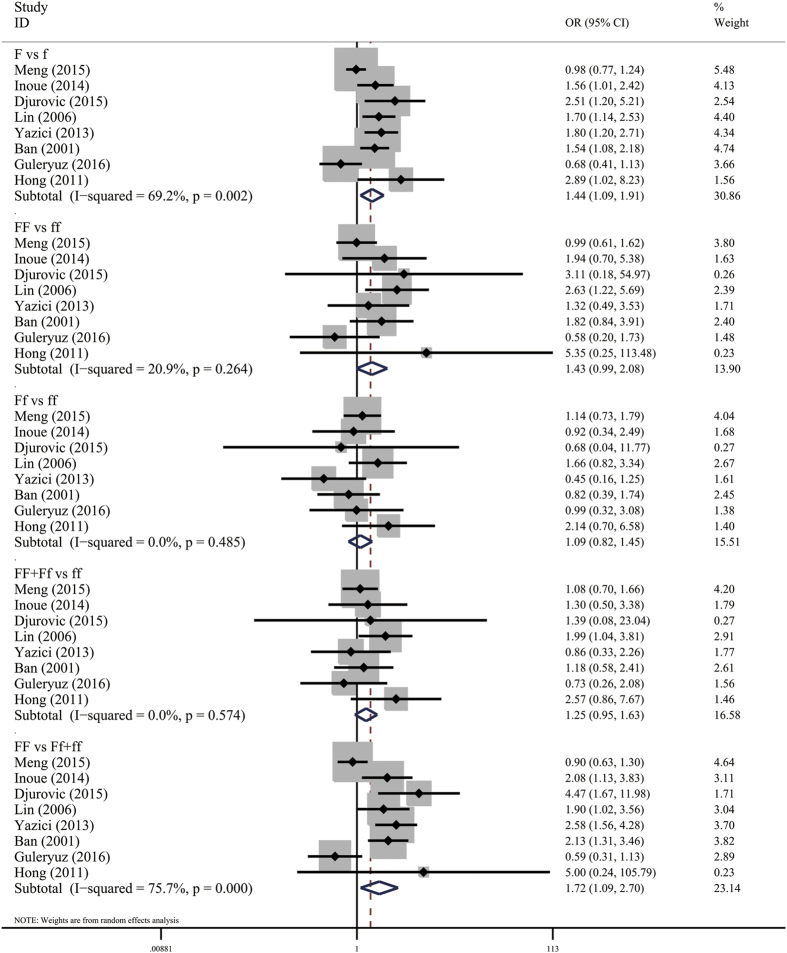
Meta-analysis of the association of *FokI* polymorphism and HT risk based on different gene models.

**Figure 3:**
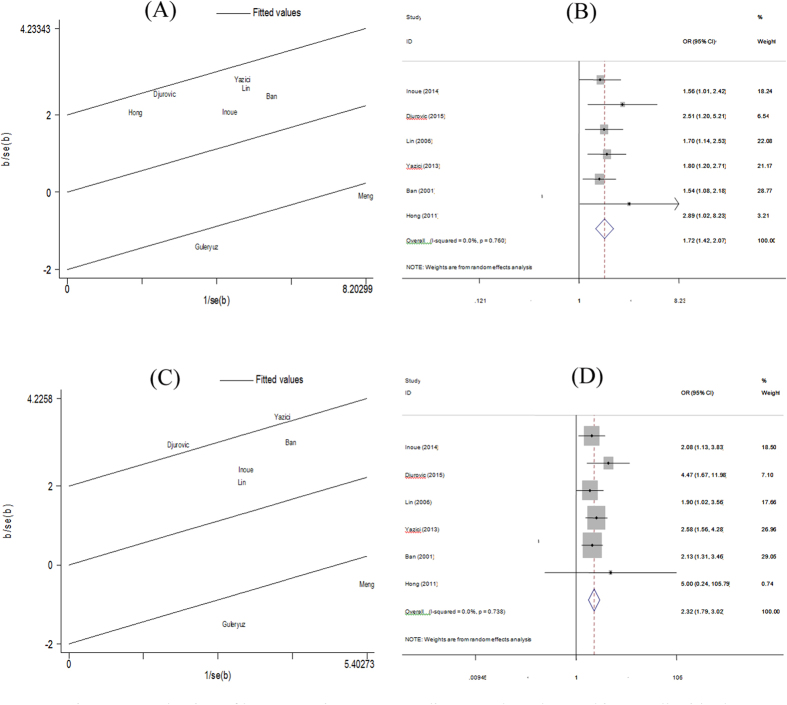
Evaluation of heterogeneity among studies on FokI polymorphism. Galbraith plot analyses for the comparisons of allele model (**A**) and recessive model (**C**); Pooled risk estimates with its 95% CIs for the allele model (**B**) and recessive model (**D**) after removing studies that contribute most to heterogeneity. b = ln(OR); se(b) = standard error of ln(OR).

Eight studies including 978 cases and 938 controls examined the association of *FokI* polymorphism and HT risk. Pooled analyses showed a significant association in the allele model (F vs f: OR = 1.44, 95% CI = 1.09–1.91, *P* = 0.010) and the dominant model (FF vs Ff + ff: OR = 1.72, 95% CI = 1.09–2.70, *P* = 0.019), but not in the other models (Table 3, Fig. 2). Significant heterogeneity existed in these two models (*I*^*2*^ = 69.2%, and *P* = 0.002 for allele model; *I*^*2*^ = 75.7%, and *P* = 0.000 for dominant model). Then, Galbraith plot analyses were performed to further explore the sources of heterogeneity. As shown in Fig. 3A and C, the studies performed by Guleryuz *et al*.^38^ and Meng *et al*.^36^ might mainly contribute to the heterogeneity. With exclusion of these studies, the heterogeneity decreased significantly (*I*^*2*^ = 0% and *P* = 0.760 for F vs f; *I*^*2*^ = 0% and *P* = 0.738 for FF vs Ff + ff) while the overall association remained significant in these two models (F vs f: OR = 1.72, 95% CI = 1.42–2.07, *P* = 0.000; FF vs Ff + ff: OR = 2.32, 95% CI = 1.79–3.02, *P* = 0.000) (Fig. 3B and D). There was one study^35^ the genotype distributions in controls departed from HWE. Sensitivity analyses by excluding this study did not change the pooled result of allele model (F vs f: OR = 1.37, 95% CI = 1.03–1.82, *P* = 0.030), but the *P* value of the dominant model was borderline (FF vs Ff + ff: OR = 1.54, 95% CI = 0.98–2.43, *P* = 0.060). Subgroup analyses by ethnicity indicated that the *FokI* F allele or FF genotype significantly increased the risk of HT in Asians (F vs f: OR = 1.45, 95% CI = 1.07–1.95, *P* = 0.016; FF vs ff: OR = 1.64, 95% CI = 1.03–2.59, *P* = 0.036; FF + Ff vs ff: OR = 1.34, 95% CI = 1.00–1.80, *P* = 0.047; FF vs Ff + ff: OR = 1.64, 95% CI = 1.03–2.64, *P* = 0.039), but the positive association was not found in Caucasians. However, significant heterogeneity were also detected in two models among studies with Asian population (F vs f: *I*^*2*^ = 63.4% and *P* = 0.027; FF vs Ff + ff: *I*^*2*^ = 65.7% and *P* = 0.020) (Table 4). Galbraith plot analyses indicated that Meng *et al*.^36^ might be the source of heterogeneity. With exclusion of this study, the pooled results remain significant (F vs F: OR = 1.64, 95% CI = 1.31–2.04, *P* = 0.000; FF vs Ff + ff: OR = 2.07, 95% CI = 1.50–2.86, *P* = 0.000), with no significant heterogeneity (F vs F: *I*^*2*^ = 0% and *P* = 0.718; FF vs Ff + ff: *I*^*2*^ = 0% and *P* = 0.940). Subgroup analyses by study quality suggested that this positive association only existed in pooled analyses of high-quality studies (F vs f: OR = 1.58, 95% CI = 1.10–2.26, *P* = 0.013; FF vs Ff + ff: OR = 1.92, 95% CI = 1.09–3.40, *P* = 0.025).

### *BsmI* polymorphism

Six studies including 837 cases and 901 controls evaluated the association of *BsmI* polymorphism and HT risk. Pooled results indicated that there was no significant correlation between *BsmI* polymorphism and HT risk in all genetic models (B vs b: OR = 0.95, 95% CI = 0.72–1.26, *P* = 0.727; BB vs bb: OR = 0.84, 95% CI = 0.46–1.52, *P* = 0.554; Bb vs bb: OR = 0.99, 95% CI = 0.76–1.29, *P* = 0.930; BB + Bb vs bb: OR = 0.96, 95% CI = 0.73–1.27, *P* = 0.764; BB vs Bb + bb: OR = 0.84, 95% CI = 0.49–1.45, *P* = 0.538) in the overall population (Table 3). Similar results were also observed in the subgroup analyses by ethnicity (Table 4). Moreover, sensitivity analyses showed the results did not change meaningfully by excluding two studies^31^,^34^ departed from HWE or one study with low-quality^36^. There was no significant heterogeneity for all models except the allele model (*I*^*2*^ = 52.1% and *P* = 0.064). A Galbraith plot analysis suggested that Stefanic *et al*.^30^ might be the source of heterogeneity for the allele model. Omitting this study, the pooled result was still not statistically significant (B vs b: OR = 1.06, 95% CI = 0.85–1.31, *P* = 0.615), with no significant heterogeneity (*I*^*2*^ = 0% and *P* = 0.621).

### *ApaI* polymorphism

Six studies including 766 cases and 813 controls evaluated the association of *ApaI* polymorphism and HT risk. The meta-analyses demonstrated no positive relationship of *ApaI* polymorphism and HT risk in the overall population (A vs a: OR = 0.98, 95% CI = 0.82–1.19, *P* = 0.869; AA vs aa: OR = 0.90, 95% CI = 0.60–1.36, *P* = 0.615; Aa vs aa: OR = 1.06, 95% CI = 0.82–1.36, *P* = 0.670; AA + Aa vs aa: OR = 1.01, 95% CI = 0.78–1.32, *P* = 0.916; AA vs Aa + aa: OR = 0.92, 95% CI = 0.65–1.29, *P* = 0.620). No significant heterogeneity was found in all the comparisons (all *P* > 0.05, Table 3). Similar results were found in the subgroup analyses by ethnicity; *ApaI* polymorphism was not associated with HT risk in Asian or Caucasian populations (Table 3). Sensitivity analyses, by excluding these two studies^33^,^35^ not in HWE or one study with low-quality^36^, suggested that the results were consistent with those of the primary analyses (all *P* > 0.05).

### *TaqI* polymorphism

A total of 902 cases and 863 controls from seven studies investigated the relationship between *TaqI* polymorphism and HT risk. The genotype distribution was consistent with HWE in the controls of all studies (all *P* > 0.05, Table 2). The pooled results showed that the *TaqI* polymorphism wasn’t significantly associated with HT risk (T vs t: OR = 1.16, 95% CI = 0.83–1.62, *P* = 0.372; TT vs tt: OR = 1.55, 95% CI = 0.87–2.76, *P* = 0.139; Tt vs tt: OR = 1.19, 95% CI = 0.79–1.81, *P* = 0.386; TT + Tt vs tt: OR = 1.42, 95% CI = 0.98–2.04, *P* = 0.064; TT vs Tt + tt: OR = 1.23, 95% CI = 0.77–1.96, *P* = 0.379, Table 3). There was significant heterogeneity for comparison of T vs t and TT vs Tt + tt (*I*^*2*^ = 70.8%, *P* = 0.002 and *I*^*2*^ = 75.4%, *P* = 0.000, respectively). In the Galbraith plots, two studies^33^,^35^ were outside of the 95%CI from the log OR, causing the heterogeneity in the results. When these two studies were excluded, the heterogeneity decreased significantly, but the pooled results were not changed significantly (T vs t: OR = 1.16, 95% CI = 0.95–1.41, P = 0.147; *I*^*2*^ = 0% and *P* = 0.635 for heterogeneity; TT vs Tt + tt: OR = 1.16, 95% CI = 0.90–1.50, P = 0.262; *I*^*2*^ = 0% and *P* = 0.788 for heterogeneity). Subgroup analyses by ethnicity found the similar results in Caucasian or in Asian (all *P* > 0.05) (Table 4).

### Publication bias

**Figure 4:**
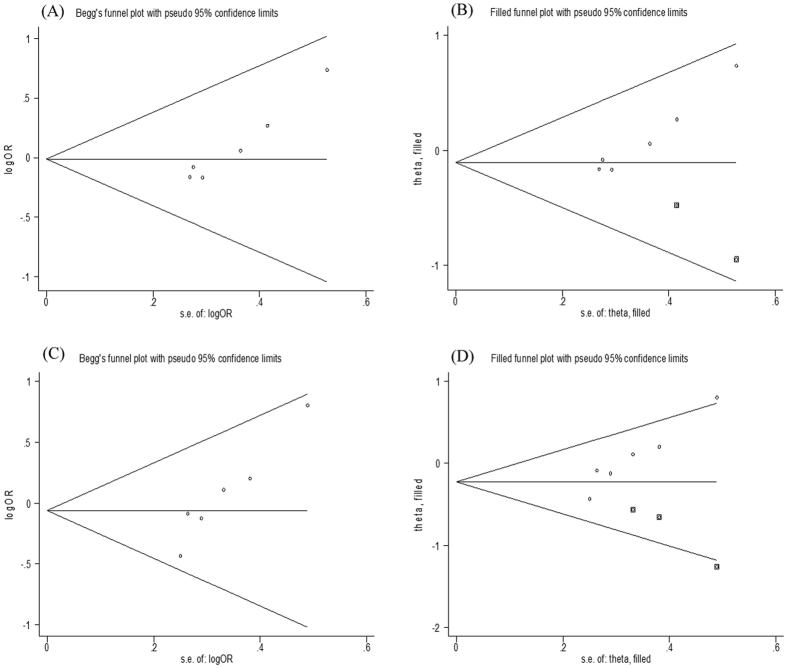
Detection of publication bias on BsmI polymorphism. Funnel plots without (**A**) and with (**B**) Trim and Fill for the analysis of Bb vs bb. Funnel plots without (**C**) and with (**D**) Trim and Fill for the analysis of BB + Bb vs bb.

No evidence of publication bias was detected by visual inspections of these funnel plots and Egger’s test in all the models regarding the *FokI, TaqI* and *ApaI* polymorphism (all *P*_Egger’s_ > 0.05). However, significant publication bias was detected in two models regarding *BsmI* polymorphism (*P*_Egger’s_ = 0.001 for Bb vs bb and *P*_Egger’s_ = 0.005 for BB + Bb vs bb) (Table 3, Fig. 4A and C). We used the trim and fill method incorporating the hypothetical studies to recalculate the pooled risk estimate. The pooled analyses continued to show no significant association between *BsmI* polymorphism and HT risk (Bb vs bb: OR = 0.90, 95% CI = 0.71–1.15, *P* = 0.397; and BB + Bb vs bb: OR = 0.80, 95% CI = 0.59–1.08, *P* = 0.141). The imputed studies produced symmetrical funnel plots (Fig. 4B and D).

## Discussion

To our knowledge, this is the first meta-analysis specially focused on the association of VDR polymorphism with HT risk. A significant association between the *BsmI* and *TaqI* polymorphisms and AITD risk has been reported by a previous meta-analysis^39^. However, in that study, the AITD, including GD and HT, was regarded as an entirety to analyze and only two studies^29^,^30^ concentrated on HT alone among all the contained studies. Although GD and HT shared similar immune-mediated mechanisms characterized by the production of thyroid autoantibodies and by thyroid lymphocytic infiltration, a number of studies has indicated that the two diseases might harbor different susceptibility genes^5^,^34^,^40^. Thus, it is necessary to perform a meta-analysis specially focused on HT. Recently, several individual studies^33^,^34^,^35^,^36^,^37^ have been conducted to investigate the association between the VDR gene polymorphisms and HT risk, but results from these studies remain conflictive and inconclusive. The reasons for this discrepancy may be small sample size, extensive geographic variations and difference in lifestyle and ethnicities. Therefore, in order to overcome the potential limitations of individual studies, we performed a meta-analysis and found that VDR *FokI* but not *TaqI, ApaI* and *BsmI* polymorphism was significantly associated with the risk of HT. Furthermore, the positive association of *FokI* polymorphism was only detected in Asians, not in Caucasians by subgroup analyses based on ethnicity.

Polymorphism *FokI* (rs2228570), located in the translational initiation site of VDR, which is the only known VDR gene polymorphism that results in the generation of an altered protein^41^,^42^,^43^. It can produce two structurally distinct isoforms: a shorter F-VDR or a longer f-VDR protein. The shorter F-VDR protein variant has been reported to be more active than the longer protein variant^44^,^45^. Transfection experiments showed the presence of short F-VDR resulted in a higher NF-kB- and NFAT-driven transcription capacity compared to the longer f-VDR. Concordantly, human monocytes and dendritic cells with a homozygous FF VDR genotype show higher expression of IL-12 (mRNA and protein) compared to the cells with an ff VDR genotype^46^. Therefore, individual with FF genotype may have a more active immune system and an increased risk to immune-mediated diseases. Eight previous studies investigated the distributional difference of *FokI* polymorphism in patients with HT and controls, and six found a positive association, but another two studies^36^,^38^ did not. By pooling these results, our meta-analysis demonstrated that the F allele might be a risk factor for susceptibility of HT (OR = 1.44, *P* = 0.010) and the incidence of HT was significantly higher in FF genotype individuals than that of Ff + ff genotype individuals in overall population (OR = 1.72, *P* = 0.019). In addition, results from subgroup analyses stratified by ethnicity indicated that HT risk was increased in Asians with FF genotype (OR = 1.64, *P* = 0.039), but not in Caucasians. This inconsistent result in these two ethnicities may be due to the influence of different genetic backgrounds, lifestyle and environment factors (such as sunlight exposure and diet). In addition, an insufficient number of samples for analysis might lead to unreliable conclusions with deviation in Caucasians.

*BsmI* (rs1544410), *ApaI* (rs7975232), and *TaqI* (rs731236) SNPs, located near the 3′ end of the VDR gene, are in strong linkage disequilibrium (LD) with each other. These three SNPs don’t change the amino acid sequence of the encoded protein but have been shown to affect gene expression through regulation of mRNA stability^47^. Three studies^30^,^33^,^38^ indicated *TaqI* polymorphism was associated with risk of HT in Croatian and Turkish population, but four other studies^34^,^35^,^36^,^37^ from China, Japan, Italy and Serbia showed no association. *ApaI* polymorphism was reported no association with HT risk in previous studies with consistent results. Regarding *BsmI* polymorphism, the study conduct by Stefanic *et al*.^30^ demonstrated B variant was apparently associated with decreased risk for HT in comparison to the reference b allele, but five other studies didn’t find this association. In present meta-analysis, pooled results showed no significant association between HT disease and *TaqI, ApaI* or *BsmI* polymorphism. Furthermore, subgroup analyses found similar results, and sensitivity analyses did not change the orientation of pooled results.

VDR 3′-RFLP haplotypes have been positioned within the regulatory element spanning-3′- untranslated region which contains polymorphic sequences affecting either VDR mRNA stability or VDR transcriptional activity^22^,^48^. Thus, *BsmI, ApaI* and *TaqI*, although functionally most likely anonymous, have been associated with total and allele-specific VDR mRNA expression^22^. Given these three variants strong LD with each other, it is rational to assess the haplotypes effects of VDR polymorphism on HT risk. Meng *et al*.^36^ reported three common haplotypes (ab, Ab and AB) of *ApaI*-*BsmI* LD block were not associated with Chinese patients with HT (*P* > 0.05). Giovinazzo *et al*.^37^ found the distribution of Bat and baT, the two most common BsmI–ApaI–TaqI haplotypes, was not significantly different in HT patients and controls from Italy. In another study^30^ conducted in Croatia, the bT and BT of *BsmI*-*TaqI* haplotypes were found to be the predisposing and protective haplotypes, respectively. Similarly, common baT as well as the rare BaT haplotypes was associated with increased and decreased risk, respectively. However, we couldn’t do meta-analysis due to insufficient published data in these studies. These effects, including effects associated with rare variants or specific stimuli need further research.

Vitamin D, well-known for its role in calcium and bone metabolism, has important effects on immune regulation by binding to the VDR localized in T lymphocytes and macrophages^49^,^50^. A number of studies^37^,^38^,^51^,^52^,^53^,^54^ have found the serum vitamin D level was lower in subjects with HT than that of healthy controls. This inverse association indicated that vitamin D deficiency might be a causal factor leading to HT. Therefore, vitamin D level might be a significant confounder which should be considered when analyzing the association of VDR and HT risk. However, a different point of view has also been postulated, which suggested that the low level of serum vitamin D seen in disease is a secondary phenomenon of VDR dysfunction rather than the reason for autoimmunity^55^. Although vitamin D level is seen as playing an important role, it is VDR dysfunction that is proposed to be the key factor in the autoimmune diseases process^56^. Because VDR is key to innate immune response which is important in the pathogenesis of autoimmune diseases^57^,^58^, VDR dysregulation greatly compromises the innate immune response. The 25-hydroxyvitamin D3 (25-OHD) level is a reliable parameter reflecting the vitamin D level of the body and usually measured as the level of vitamin D. When VDR dysregulation, the expression of CYP24A1, an enzyme that inactivating 1,25-dihydroxyvitamin D (1,25-OHD) was inhibited. Increased 1,25-OHD will decrease 25-OHD by reducing gene expression and inhibiting expression of CYP27A1 which is an enzyme involved in conversion of vitamin D into 25-OHD^55^,^59^. Among our included studies, only two studies concurrently provided the information on vitamin D levels and VDR in patients with HT. One study^37^ found that the prevalence of vitamin D deficiency in HT patients was significantly higher than that in the control group (70% vs 18.2%; *P* = 0.0001), but VDR *BsmI, ApaI*, and *TaqI* polymorphisms were not associated with HT risk. The other^38^ indicated that the prevalence of vitamin D insufficiency in HT cases was significantly higher than controls (*P* = 0.02) while VDR *TaqI*, but not *FokI* polymorphisms is associated with HT. It is unfortunate that neither study analyzed the distributional difference of VDR polymorphisms stratified by vitamin D levels. Therefore, the mechanism and effect for the interaction of vitamin D and VDR in patients with HT need further investigations.

Several limitations should be discussed when explaining the results of our meta-analysis. First, lack of adjustments for some factors, such as age, gender, thyroid functional status, circulating vitamin D levels, or dietary vitamin D intake, which may influence the association between VDR variants and risk of HT, might bias the present results. Second, because of unpublished data or limited number of studies, significant publication bias was found in two models regarding *BsmI* polymorphism, which might have some impact on the final outcome. However, we used trim and fill method to assess the influence of publication bias and found that the results were not significantly changed with or without the addition of hypothetical missing studies. Heterogeneity among studies was also detected in some analyses due to ethnic difference, geographic characteristics and lifestyle. However, our sensitivity analysis showed that studies that contribute to heterogeneity did not significantly alter the conclusions of the overall OR. Third, the statistical power to detect the association may be lower because number of studies included in our meta-analysis is relatively small. However, Ioannidis *et al*.^60^ estimated the median sample size required to detect the observed summary effects in each population addressed in 752 studies is 3,535, which is 13.3-fold more subjects than in each original study. These sample size requirements can be inflated considerably if trying to account for potential bias or heterogeneity. These estimates may be difficult to address even by very large biobanks and observational cohorts. Therefore, meta-analysis is an effective way to explore the truth before the emergence of large sample data. Further studies should be focusing on innovative study designs and strong collaborative efforts.

In conclusion, our meta-analysis suggests that the VDR *FokI* polymorphism is associated with HT risk in overall population or in Asians, but not in Caucasians. The *TaqI, ApaI* and *BsmI* polymorphisms are not associated with HT risk. Further well-designed studies with larger sample sizes and different ethnic population are needed to clarify the present findings. Furthermore, the exact causality and mechanism for the interaction of VDR and HT development need further experimental or animal mechanism studies.

## Methods

### Search strategy

We identified all the studies regarding the relationship of VDR gene polymorphisms and HT by searching PubMed, Embase, China National Knowledge Internet (CNKI), and Wan fang databases without language restrictions (the last search update performed on September 30, 2016). The following key words and search terms were used to identify relative publications: “Vitamin D receptor”, “VDR”, “*ApaI*”, “*BsmI*”, “*FokI*”, “*TaqI*” and “hashimoto’s thyroiditis”. The reference lists of identified articles and related reviews were reviewed for additional studies.

### Inclusion and exclusion criteria

Studies meeting all of the following inclusion criteria were included: (1) case-control study or cohort study; (2) investigating the association between VDR gene polymorphisms (*ApaI, BsmI, FokI* and *TaqI*) and HT risk; and (3) providing the frequencies of the variants in cases and controls or providing sufficient data to calculate the estimation of odds ratios (ORs) with 95% confidence interval (95% CI). Exclusion criteria were as follows: (1) overlapping data; (2) studies without genotype frequency and genotype distribution or insufficient information for data extraction; (3) family-based study design; and (4) abstracts, reviews, comments or editorial articles lack of necessary raw data. In the case of overlapping data, only the study with the largest population was selected for this meta-analysis.

### Data extraction

Two investigators (XF Wang and WL Cheng) extracted data independently. Any disagreement was resolved through discussion. The extracted data included: name of the first author, year of publication, country, ethnicity, number of cases and controls, genotyping method, control sources, and genotype distribution in cases and controls.

### Quality Assessment

The quality of included studies was assessed by two independent reviewers (XF Wang and Y Ma) using the Newcastle-Ottawa Scale (NOS)^61^. The NOS judged a study based on three perspectives: selection, comparability and exposure/outcome. The full score was 9 stars. Study that scored above six stars was considered as high quality.

### Statistical analysis

A random-effects model was used to incorporate within- and between-study heterogeneity as this can provide more conservative result than a fixed effects model^62^. Pooled ORs and their respective 95% CIs were calculated to evaluate the association between the four VDR SNPs and HT risk under five genetic models: the allele model (eg, A vs a), the homozygous model (eg, AA vs aa), the heterozygous model (eg, Aa vs aa), the recessive model (eg, AA + Aa vs aa), and the dominant model (eg, AA vs Aa + aa). The Hardy-Weinberg equilibrium (HWE) in controls was tested using the goodness-of-fit χ2 statistic with one degree of freedom^63^. Cochrane’s Q test and *I*^*2*^ test were used to assess heterogeneity among trials. Q-test reported a *P* value < 0.1 or *I*^*2*^ > 50% was defined as significant heterogeneity^64^. In case of substantial heterogeneity, a Galbraith plot was created to graphically identify the potential outlier studies that might cause the heterogeneity. Then, a meta-analysis was rerun after excluding the outlier studies^65^. Subgroup analyses were performed based on ethnicity and quality of included studies to avoid the potential bias influence. Sensitivity analyses were performed by excluding each individual study or the studies with controls inconsistent with HWE to evaluate the impact of individual study on the pooled risk estimate. Publication bias was evaluated by a visual inspection of funnel plot and Egger’s test^66^. If publication bias was indicated, the “trim and fill” method which conservatively imputes hypothetical negative unpublished studies to mirror the positive studies that cause funnel plot asymmetry was performed to further assess the possible effect of publication bias^67^. All *P*-values were two-tailed. All analyses were performed using Stata 11.0 (Stata Corporation, College Station, TX, USA). This article follows the PRISMA statement^68^ and the Cochrane Collaboration guidelines for reporting meta-analysis.

## Additional Information

**How to cite this article**: Wang, X. *et al*. Vitamin D receptor gene FokI but not TaqI, ApaI, BsmI polymorphism is associated with Hashimoto’s thyroiditis: a meta-analysis. *Sci. Rep.* **7**, 41540; doi: 10.1038/srep41540 (2017).

**Publisher's note:** Springer Nature remains neutral with regard to jurisdictional claims in published maps and institutional affiliations.

## Figures and Tables

**Table 1:** Studies characteristics of each article included in the meta-analysis.

Study	Year	Country	Ethnicity	Genotyping method	Control sources	Sample size (case/control)	Age (case/control)	% Female (case/control)	SNPs	Matched factors	NOS score (*)
Ban^28^	2001	Japan	Asian	PCR-RFLP	NR	130/150	NR/NR	100/100	*FokI*	NR	6
Lin^29^	2006	China	Asian	PCR-RFLP	PB	109/90	36 ± 12/NR	89.9/NR	*FokI*	Region	7
Stefanic^30^	2008	Croatia	Caucasian	PCR-RFLP	PB	145/145	44 ± 14/42 ± 14	93.1/93.1	*TaqI, ApaI, BsmI*	Age, sex, ethnicity, region	9
Huo^31^	2010	China	Asian	PCR-RFLP	PB	115/120	38 ± 13/37 ± 6.2	80.9/75.0	*BsmI*	Region	8
Hong^32^	2011	China	Asian	PCR-RFLP	PB	82/80	NR/NR	64.6/75.0	*FokI*	NR	7
Yazici^33^	2013	Turkey	Caucasian	PCR-RFLP	PB	111/159	48 ± 13/31 ± 6.3	86.8/95.5	*TaqI, ApaI, FokI*	NR	7
Inoue^34^	2014	Japan	Asian	PCR-RFLP	PB	116/76	NR/28.9 ± 11	NR/64.5	*TaqI, ApaI, FokI, BsmI*	NR	7
Djurovic^35^	2015	Serbia	Caucasian	PCR-RFLP	PB	44/32	38 ± 5.4/NR	100/100	*TaqI, ApaI, FokI*	Age, sex, region	9
Meng^36^	2015	China	Asian	MALDI-TOF-MS	HB	250/301	31.9 ± 13/33.6 ± 13	84.4/69.8	*TaqI, ApaI, FokI, BsmI*	NR	6
Giovinazzo^37^	2016	Italy	Caucasian	PCR-RFLP	PB	100/100	42 ± 15/40 ± 13	87/88	*TaqI, ApaI, BsmI*	Age, sex, region	9
Guleryuz^38^	2016	Turkey	Caucasian	PCR-RFLP	PB	136/50	39 ± 9.9/35 ± 11	91.2/90.0	*TaqI, FokI*	Sex	8

MALDI-TOF-MS: Matrix assisted laser desorption ionization-time of flight mass spectrometer; PCR-RFLP: Polymerase chain reaction–restriction fragment length polymorphism; PB: Population-based; HB: Hospital-based; NR: Not reported; NOS, Newcastle-Ottawa Scale.

**Table 2:** Distribution of VDR genotype and allele in Hashimoto’s thyroiditis patients and controls.

Study	Year	Genotype distribution in case			Genotype distribution in control			*P* value of distribution	*P* value of HWE
*FokI* (rs2228570)		FF (CC)	Ff (CT)	ff (TT)	FF (CC)	Ff (CT)	ff (TT)		
Ban^28^	2001	64	51	15	47	83	20	0.008	0.078
Lin^29^	2006	40	48	21	21	40	29	0.046	0.324
Hong^32^	2011	2	10	70	0	5	75	0.099	0.773
Yazici^33^	2013	75	28	8	71	78	10	0.000	0.058
Inoue^34^	2014	54	43	10	25	42	9	0.060	0.172
Djurovic^35^	2015	28	15	1	9	22	1	0.008	0.008
Meng^36^	2015	75	129	46	97	145	59	0.725	0.716
Guleryuz^38^	2016	61	57	18	29	16	5	0.282	0.234
*BsmI* (rs1544410)		BB (AA)	Bb (AG)	bb (GG)	BB (AA)	Bb (AG)	bb (GG)		
Stefanic^30^	2008	20	69	56	42	61	42	0.006	0.056
Huo^31^	2010	2	9	69	1	7	112	0.241	0.035
Yazici^33^	2013	16	58	37	24	85	50	0.946	0.214
Inoue^34^	2014	4	21	73	3	11	50	0.795	0.042
Meng^36^	2015	1	22	227	0	31	270	0.383	0.346
Giovinazzo^37^	2016	37	40	23	34	41	25	0.895	0.083
*ApaI* (rs7975232)		AA (TT)	Aa (TG)	aa (GG)	AA (TT)	Aa (TG)	aa (GG)		
Stefanic^30^	2008	32	83	30	42	80	23	0.312	0.139
Yazici^33^	2013	35	58	18	39	100	20	0.218	0.001
Inoue^34^	2014	7	49	51	12	32	31	0.118	0.445
Djurovic^35^	2015	20	14	10	12	8	12	0.373	0.005
Meng^36^	2015	18	104	128	20	113	168	0.556	0.865
Giovinazzo^37^	2016	31	53	16	35	45	20	0.512	0.428
*TaqI* (rs731236)		TT (TT)	Tt (TC)	tt (CC)	TT (TT)	Tt (TC)	tt (CC)		
Stefanic^30^	2008	60	70	15	51	66	28	0.092	0.426
Yazici^33^	2013	66	36	9	44	90	25	0.000	0.061
Inoue^34^	2014	87	28	1	58	17	0	0.585	0.268
Djurovic^35^	2015	20	14	3	24	7	1	0.180	0.591
Meng^36^	2015	224	24	2	266	34	1	0.622	0.938
Giovinazzo^37^	2016	38	42	20	30	49	21	0.471	0.904
Guleryuz^38^	2016	62	56	18	23	19	7	0.954	0.356

HWE: Hardy-Weinberg equilibrium.

**Table 3:** Meta-analyses of the association between VDR gene polymorphisms and Hashimoto’s thyroiditis risk.

SNPs	Sample size* (case/control)	Genetic models	Test for association		Test for heterogeneity		*P*_Egger’s test_
			OR (95% CI)	*P*	*I*^*2*^ (%)	*P*	
*FokI* rs2228570 (*n* = 8)	978/938	F vs f	1.44 (1.09–1.91)	**0**.**010**	69.2	**0**.**002**	0.158
		FF vs ff	1.43 (0.99–2.08)	0.059	20.9	0.264	0.526
		Ff vs ff	1.09 (0.82–1.45)	0.566	0	0.485	0.594
		FF + Ff vs ff	1.25 (0.95–1.63)	0.107	0	0.574	0.793
		FF vs Ff + ff	1.72 (1.09–2.70)	**0**.**019**	75.7	**0**.**000**	0.290
*BsmI* rs1544410 (*n* = 6)	837/901	B vs b	0.95 (0.72–1.26)	0.727	52.1	0.064	0.121
		BB vs bb	0.84 (0.46–1.52)	0.554	43.5	0.115	0.380
		Bb vs bb	0.99 (0.76–1.29)	0.930	0	0.672	**0**.**001**
		BB + Bb vs bb	0.96 (0.73–1.27)	0.764	18.5	0.293	**0**.**005**
		BB vs Bb + bb	0.84 (0.49–1.45)	0.538	45.9	0.100	0.545
*ApaI* rs7975232 (*n* = 6)	766/813	A vs a	0.98 (0.82–1.19)	0.869	33.2	0.187	0.896
		AA vs aa	0.90 (0.60–1.36)	0.615	33.2	0.187	0.999
		Aa vs aa	1.06 (0.82–1.36)	0.670	5.7	0.380	0.438
		AA + Aa vs aa	1.01 (0.78–1.32)	0.916	18.3	0.295	0.607
		AA vs Aa + aa	0.92 (0.65–1.29)	0.620	37.4	0.157	0.719
*TaqI* rs731236 (*n* = 7)	902/863	T vs t	1.16 (0.83–1.62)	0.372	70.8	**0**.**002**	0.052
		TT vs tt	1.55 (0.87–2.76)	0.139	40.9	0.118	0.147
		Tt vs tt	1.19 (0.79–1.81)	0.386	0	0.687	0.208
		TT + Tt vs tt	1.42 (0.98–2.04)	0.064	0	0.440	0.130
		TT vs Tt + tt	1.23 (0.77–1.96)	0.379	75.4	**0**.**000**	0.113

^*^Sample size refers to the total number of genotype for cases and controls; *n* number of involved studies; Bold indicating *P* < 0.05.

**Table 4:** Subgroup analyses of the association between VDR gene polymorphisms and Hashimoto’s thyroiditis risk based on ethnicity.

SNPs	Ethnicity	Sample size* (**case/control**)	Genetic model	Test for association		Test for heterogeneity	
				OR (95%CI)	*P*	*I*^*2*^ (%)	*P*
***FokI*** **rs2228570**	Asian (*n* = 5)	687/697	F vs f	**1**.**45** (**1**.**07**–**1**.**95**)	**0**.**016**	63.4	0.027
			FF vs ff	**1**.**64** (**1**.**03**–**2**.**59**)	**0**.**036**	32.6	0.204
			Ff vs ff	1.19 (0.88–1.62)	0.264	0	0.535
			FF + Ff vs ff	**1**.**34** (**1**.**00**–**1**.**80**)	**0**.**047**	0	0.421
			FF vs Ff + ff	**1**.**64** (**1**.**03**–**2**.**64**)	**0**.**039**	65.7	0.020
	Caucasian (*n* = 3)	291/241	F vs f	1.42 (0.67–3.00)	0.358	82.8	0.003
			FF vs ff	0.98 (0.49–2.00)	0.964	0	0.397
			Ff vs ff	0.64 (0.31–1.34)	0.239	0	0.598
			FF + Ff vs ff	0.83 (0.42–1.64)	0.586	0	0.907
			FF vs Ff + ff	1.84 (0.59–5.73)	0.296	87.8	0.000
***BsmI*** **rs1544410**	Asian (*n* = 3)	481/497	B vs b	1.15 (0.79–1.67)	0.472	1.2	0.363
			BB vs bb	1.52 (0.46–5.05)	0.816	0	0.585
			Bb vs bb	0.97 (0.61–1.56)	0.924	0	0.389
			BB + Bb vs bb	1.20 (0.58–2.14)	0.473	25.9	0.259
			BB vs Bb + bb	1.44 (0.44–4.79)	0.550	0	0.573
	Caucasian (*n* = 3)	356/404	B vs b	0.85 (0.59–1.22)	0.377	67.8	0.045
			BB vs bb	0.84 (0.46–1.52)	0.499	68.3	0.043
			Bb vs bb	0.92 (0.66–1.28)	0.622	0	0.886
			BB + Bb vs bb	0.83 (0.61–1.14)	0.254	0	0.383
			BB vs Bb + bb	0.75 (0.38–1.46)	0.394	71.6	0.030
***ApaI*** **rs7975232**	Asian (*n* = 2)	366/377	A vs a	0.92 (0.58–1.49)	0.744	70.8	0.064
			AA vs aa	0.69 (0.21–2.23)	0.537	72.6	0.056
			Aa vs aa	1.14 (0.84–1.54)	0.418	0	0.479
			AA + Aa vs aa	1.04 (0.69–1.56)	0.865	37.7	0.205
			AA vs Aa + aa	0.68 (0.23–1.94)	0.466	69.1	0.072
	Caucasian (*n* = 4)	400/436	A vs a	1.00 (0.79–1.25)	0.965	25.9	0.256
			AA vs aa	0.96 (0.61–1.52)	0.869	18.8	0.297
			Aa vs aa	0.99 (0.62–1.59)	0.978	31.0	0.226
			AA + Aa vs aa	0.99 (0.64–1.53)	0.973	29.7	0.234
			AA vs Aa + aa	0.99 (0.69–1.42)	0.950	29.1	0.237
***TaqI*** **rs731236**	Asian (*n* = 2)	366/377	T vs t	0.98 (0.66–1.46)	0.935	0	0.596
			TT vs tt	0.45 (0.07–3.10)	0.413	0	0.934
			Tt vs tt	0.41 (0.06–2.93)	0.376	0	0.836
			TT + Tt vs tt	0.45 (0.07–3.06)	0.411	0	0.918
			TT vs Tt + tt	1.03 (0.67–1.57)	0.895	0	0.567
	Caucasian (*n* = 5)	536/486	T vs t	1.16 (0.83–1.62)	0.346	77.7	0.001
			TT vs tt	1.55 (0.87–2.76)	0.085	51.0	0.086
			Tt vs tt	1.24 (0.83–1.85)	0.288	0	0.606
			TT + Tt vs tt	1.47 (0.99–2.19)	0.058	9.2	0.354
			TT vs Tt + tt	1.31 (0.70–2.48)	0.402	81.4	0.000

^*^Sample size refers to the total number of genotype for cases and controls; *n* number of involved studies; Bold indicating *P* < 0.05.
